# Addressing social determinants of health as effective readmission prevention and discharge support

**DOI:** 10.1002/jgf2.70059

**Published:** 2025-08-27

**Authors:** Kakeru Iwase, Yuya Yokota, Shintaro Kosaka, Kazushige Fujiwara, Junki Mizumoto

**Affiliations:** ^1^ Aoga‐Shima Clinic Aogashima, Tokyo Japan; ^2^ Department of General Internal Medicine Okayama University Hospital Okayama Japan; ^3^ Oomagari Clinic Izumo, Shimane Japan; ^4^ Department of Hospital General Medicine Tokyo Metropolitan Hiroo Hospital Shibuya, Tokyo Japan; ^5^ Department of Family Practice Ehime Seikyo Hospital Matsuyama, Ehime Japan

To the Editor,

Effective collaboration both within and beyond hospital settings, grounded in an understanding of the social determinants of health (SDH), can improve the quality of care, especially in discharge planning and readmission prevention.^1^ In acute care hospital wards, where medical services are often fragmented, general practitioners are expected to deliver care informed by SDH‐related evidence, adopting a holistic and patient‐centered approach.

At the 30th Annual Meeting of the Japanese Society of Hospital General Medicine, we convened a symposium to explore the necessity of evidence‐based interventions targeting SDH to reduce readmissions and support effective discharge planning in hospital settings.

First, we presented recent evidence on the prevention of readmissions through interventions addressing the social determinants affecting patients with chronic heart failure—an ambulatory care‐sensitive condition of great relevance. While the evidence on the effectiveness of social interventions for reducing readmissions remains inconsistent, current data suggest that individualized, context‐sensitive support may provide benefit. For instance, younger male patients have been identified as being at higher risk of readmission, and targeted lifestyle modifications, including dietary changes and medication adherence, have demonstrated efficacy.^2^ Additionally, a retrospective observational study of heart failure patients in Japan shows that structured once‐weekly monitoring for patients with dementia is independently associated with readmission, demonstrating the importance of social support.^3^

Second, we introduced a case study from a large acute care hospital that has implemented a continuum of care framework focused on the “Patient Journey.” This initiative seeks to identify patients' SDH and deliver context‐sensitive support aligned with their personal values. We demonstrated that screening for SDH during hospitalization, alongside integrated admission and discharge planning, can improve post‐discharge outcomes for both older adults and pediatric patients.^4^ Among older patients, a narrative review and thematic analysis highlight the complexity of frail syndrome and the need for contextualized interventions.^5^ By embedding SDH screening into the clinical pathways for geriatric syndromes, socioeconomic status, educational attainment, social support, and housing conditions were systematically addressed by the interdisciplinary admission and discharge support team. This approach has the potential to improve healthcare quality, including reduced hospital length of stay and lower readmission rates.

Finally, we discussed an original case that one of the authors experienced involving a heart failure patient who experienced fragmented care delivery, and we examined strategies for the patient journey. The case is an 80‐year‐old woman with chronic heart failure and mild dementia living alone in a mountainous area of Japan. She was hospitalized for heart failure without the clinic doctor's knowledge, and her heart failure worsened again after discharge because of lack of cooperation with the hospital from which she was admitted. This case prompted critical discussion among the audience. Hospitalization presents a critical opportunity to address social frailty, initiate dementia assessments, and facilitate the introduction of long‐term care insurance services. It is essential to involve all relevant stakeholders who contribute to supporting the patient's daily life and long‐term well‐being.

In conclusion, emphasizing SDH and fostering collaborative care across institutional boundaries can substantially improve healthcare quality. As optimal intervention strategies vary based on individual patient contexts and conditions, it is imperative that general practitioners and hospital‐based primary care physicians continue to actively publish case studies and empirical research to guide future practice.

## CONFLICT OF INTEREST STATEMENT

Dr. Shintaro Kosaka has received honoraria for lectures from Otsuka Pharmaceutical Factory and Mypecon, and honoraria for manuscript writing from Cardinal Health, MEDSI, and IGAKU‐SHOIN Ltd. He has also received research funding from Triple W Japan and the Health Labour Sciences Research Grant. All other authors declare that they have no conflicts of interest.

## Data Availability

The data that supports the findings of this study are available in the supplementary material of this article.
